# A Comprehensive Review of the Diagnosis and Management of Acute Liver Failure

**DOI:** 10.3390/jcm12237451

**Published:** 2023-11-30

**Authors:** Nazli Begum Ozturk, Emre Herdan, Fuat H. Saner, Ahmet Gurakar

**Affiliations:** 1 Department of Internal Medicine, Beaumont Hospital, Royal Oak, MI 48073, USA; 2 Division of Gastroenterology and Hepatology, Johns Hopkins University Hospital, Baltimore, MD 21287, USA; 3 Department of General, Visceral and Transplantation Surgery, University of Duisburg-Essen, 45147 Essen, Germany; 4 Organ Transplant Center of Excellence, King Faisal Specialized Hospital & Research Center, Riyadh 11211, Saudi Arabia

**Keywords:** liver disease, hepatic encephalopathy, acetaminophen, fulminant hepatitis, intensive care

## Abstract

Acute liver failure (ALF) is a rare and specific form of severe hepatic dysfunction characterized by coagulopathy and hepatic encephalopathy in a patient with no known liver disease. ALF carries a high morbidity and mortality. Careful attention should be given to hemodynamics and metabolic parameters along with the active surveillance of infections. Timely transfer and supportive management are important in an intensive care unit in a liver transplant center. Identifying patients who will and will not improve with medical management and may need emergent liver transplantation is critical. In this review, we provide a comprehensive update on the etiology, diagnosis, and management of ALF.

## 1. Introduction

Acute liver failure (ALF) refers to the development of severe hepatic dysfunction characterized by an international normalized ratio (INR) of ≥1.5 and altered mental status due to hepatic encephalopathy (HE) in a patient with no known liver disease. ALF is also referred to as fulminant hepatitis, acute hepatic necrosis, and fulminant hepatic necrosis. ALF is defined when the interval between the onset of jaundice and the development of HE is <26 weeks per the American Association for the Study of Liver Diseases (AASLD) and American College of Gastroenterology guidelines [1,2]. ALF is often incorrectly used in the setting of an acute episode of liver dysfunction in patients with chronic liver disease, such as acute-on-chronic liver failure (ACLF), or other systemic diseases affecting the liver [3]. In exceptional cases, ALF can be present in patients with unrecognized Wilson disease, reactivation of chronic hepatitis B infection, de novo presentation of autoimmune hepatitis, or Budd–Chiari syndrome, even though chronic liver disease or advanced fibrosis can be present in these patients [3,4]. To define ALF in these patients, the duration of illness must be <26 weeks. Apart from this, patients with severe alcohol-related hepatitis are considered as ACLF, not ALF, even if the duration of illness is <26 weeks, as the majority of these patients have a history of heavy alcohol use. If INR >1.5 is present without HE, and the ALT level is >10 times the upper limit of normal value, the condition is defined as acute liver injury (ALI) as ALF diagnosis requires the presence of both coagulopathy and HE [3]. Secondary causes of coagulopathy, elevated liver tests, and altered mental status should be investigated, and if no primary liver etiology is identified, a systemic disease process should be considered. If left untreated, ALF carries a poor prognosis with a mortality rate of 30–50% [4,5].

## 2. Causes of Acute Liver Failure

Drug-induced liver injury (DILI) is the primary cause of ALF in the United States of America (USA), Europe, and Australia, while acute hepatitis A virus (HAV), hepatitis B virus (HBV), and hepatitis E virus (HEV) are the primary causes in Asia and Africa [4,5]. Acetaminophen is the most common medication associated with ALF and is responsible for 45.7% of ALF cases in North America, and 65.4% in the UK [6]. In Europe, it is reported that 8% of all liver transplantations (LT) are performed due to ALF, and of those ALF cases, 19% are caused by viral hepatitis, 18% by DILI, 4% have toxic causes, 3% have traumatic or post-operative ALF, while 56% have unknown or other causes [7]. In India, viral hepatitis (HAV, HBV, HEV) is the primary cause of ALF, followed by DILI, mainly due to antituberculosis drugs [8,9]. In China, traditional Chinese medicines, herbal and dietary supplements, and antituberculosis drugs have been identified as the leading causes of DILI [10].

Liver injury caused by acetaminophen overdose is usually characterized by extreme hepatocellular pattern liver transaminase elevations with normal or slightly increased bilirubin levels within 8–12 h of excess acetaminophen intake, and often progresses rapidly to multiorgan failure and coma. Multiorgan failure with acetaminophen overdose is due to an initial massive proinflammatory response causing systemic inflammatory response syndrome (SIRS) and a compensatory anti-inflammatory response leading to immune cell dysfunction [11]. Lactic acidosis, acute kidney injury (AKI), hypoglycemia, and hypophosphatemia can also be seen in patients with ALF due to acetaminophen overdose. Severe metabolic acidosis may require renal replacement therapy (RRT) in certain patients. Other causes of metabolic acidosis such as salicylates, tricyclic antidepressants, or methanol overdose should also be investigated.

Less than 1% of the patients with HAV infection develop ALF, and ALF is mainly seen in elderly patients, who generally have a worse prognosis. Overall, ALF due to HAV has a good prognosis with 70% spontaneous or transplant-free survival (4). HBV is the most common viral cause of severe ALF due to either de novo infection, reactivation in a patient with previous HBV infection, or delta superinfection [3]. Less than 4% of patients with acute HBV develop ALF. Mortality rates for ALF due to HBV are higher than for ALF due to HAV or HEV infections. Acute hepatitis C virus (HCV) infection very rarely progresses to ALF. Herpes simplex virus 1 and 2 (HSV1, HSV2) and varicella-zoster virus (VZV) are rare causes of ALF, and are more commonly seen in immunosuppressed patients; however, ALF may occur in immunocompetent patients. Epstein–Barr virus (EBV) and cytomegalovirus (CMV) nucleic acid tests should be performed in patients with no clear etiology. Skin or mucous membrane vesicles may be seen with HSV.

In the USA and Europe, approximately 8% of all cases of ALF are due to HBV. About two-thirds of ALF cases due to HBV are because of new infections and the remainder are due to reactivation of HBV in the setting of unrecognized chronic HBV infection or in the setting of immunosuppression, such as chemotherapy or immune modulators [4]. ALF due to reactivation of HBV usually has a worse prognosis than that caused by de novo HBV infection. Acute HEV is mostly seen in patients with a travel history to endemic areas, such as Asia and parts of Africa, although very rarely it can be seen in Europe and in the USA. Mortality rates for ALF due to HEV are low; however, worse outcomes have been reported in elderly patients, those with preexisting chronic liver disease, and pregnant women.

ALF due to non-acetaminophen DILI often presents with less severe transaminase elevations and higher bilirubin levels compared to acetaminophen, and multiorgan failure is less common. The most common causes of non-acetaminophen toxicity are antituberculosis drugs (mainly isoniazid), antimicrobials (nitrofurantoin and ketoconazole), antiepileptics (valproic acid, phenytoin), and non-steroidal anti-inflammatory drugs (NSAIDs) [3]. Non-acetaminophen DILI is usually seen within six months of drug initiation and often presents with lower aminotransferase levels but higher bilirubin concentrations compared to acetaminophen or ischemic hepatitis. Idiosyncratic drug reactions are rare and not dose-dependent. If non-acetaminophen DILI progresses to ALF, which occurs in approximately 10% of patients, up to 70–80% of the latter need emergent LT or die [12,13]. Mushroom poisoning, mainly with amanita phalloides and related species, usually presents with severe abdominal pain, nausea, vomiting, and diarrhea within hours to one day of ingestion and acute renal failure usually precedes ALF [14].

If any other autoimmune disease is present, suspicion for autoimmune hepatitis (AIH) as the cause of ALF should be considered. Approximately 80% of patients with ALF due to AIH are women and ALF due to AIH has particularly poor outcomes and often requires LT [4,15]. Patients with AIH often have elevated IgG levels, positive anti-smooth muscle antibody (ASMA), antinuclear antigen (ANA), liver–kidney microsomal antibody (LKM), or any combination of these. It should be noted that those antibodies can be negative in the setting of ALF due to AIH, and their absence does not rule out AIH. In addition, mildly positive antibodies can also be present in other conditions causing ALF. Liver biopsy may help in the diagnosis of ALF due to AIH; however, it often lacks the typical AIH findings of portal inflammation with plasma-cell-rich infiltrate and interface hepatitis, but rather shows centrilobular hemorrhagic necrosis preceding chronic portal inflammation [3]. With the progression of ALF, necrosis can become confluent, making the histological diagnosis challenging.

In ALF due to Wilson disease, most patients are <20 years of age, and it presents with Coombs-negative hemolytic anemia and high bilirubin to ALP ratio. ALP is usually normal or very low. Approximately half of the patients have Kayser–Fleischer rings; however, it may be difficult to assess this finding in patients with HE. In addition, most patients also rapidly progress to renal failure and have low serum uric acid levels. Serum ceruloplasmin can be very low but can also be normal or increased in ALF. Falsely normal serum ceruloplasmin can be seen in Wilson disease as it is an acute phase reactant. In addition, serum ceruloplasmin can also be low in cases of ALF due to other causes. Serum and urinary copper are increased; however, their values are not reliable in the setting of chronic liver disease. Twenty-four-hour urine copper excretion may also be unreliable in the setting of kidney dysfunction and low urine output. Liver biopsy to estimate liver copper is also not reliable in the setting of ALF, in particular in the setting of cholestasis. Genetic studies of ATP7B testing results are often not available on short notice at most centers. Patients with ALF due to Wilson disease rarely recover without transplantation.

Pregnancy-related ALF occurs in the third trimester in the form of hemolysis, elevated liver enzymes, low platelet count (HELLP) syndrome, or acute fatty liver of the pregnancy (AFLP) [16]. AFLP is characterized by diffuse hepatic steatosis, low transaminase levels, hypoglycemia, and increased urate levels, and has a 20% mortality rate. Immediate delivery of the fetus is needed in both HELLP syndrome and AFLP, and emergent LT is rarely needed.

ALF after hemi-hepatectomy can be seen after an extensive resection of the liver. Spontaneous recovery is usually seen if there is no preexisting chronic liver disease. The AST/ALT ratio is usually >1 in Budd–Chiari syndrome, and it is most commonly seen in women in their fourth or fifth decades and is associated with hypercoagulable states [17]. Anticoagulation should be initiated as soon as a diagnosis is made and transjugular intrahepatic portosystemic shunt (TIPS) is the preferred intervention for decompression in parallel with LT listing if there is no response to therapeutic interventions [2]. Survival rates have been reported as 37–40%, despite LT. Secondary causes of ALF may include sepsis, malaria, leptospirosis, rickettsial disease, thyroid disease, Still’s disease, and hemophagocytic syndromes [3,18]. Despite extensive investigation in many patients, the cause of ALF remains unknown [5]. Common causes of ALF are shown in Table 1, and a special pattern of liver injury is shown in Table 2.

## 3. Clinical Manifestations

Many symptoms of ALF are unspecific and may include fatigue, lethargy, confusion, generalized weakness, nausea, right upper quadrant pain, jaundice, and pruritus. Physical examination findings may reveal right upper quadrant tenderness, hepatomegaly, ascites, and vesicular skin lesions with HSV infection. Symptoms of cognitive dysfunction may not be overt on presentation. Laboratory test abnormalities often include elevated liver tests (often markedly elevated), and elevated bilirubin level. INR ≥ 1.5 is part of the ALF definition and must be present. Other laboratory findings, such as hemolytic anemia, elevated serum creatine and blood urea nitrogen, hypoglycemia, hypophosphatemia, hypomagnesemia, elevated amylase and lipase, elevated ammonia level, and elevated lactate dehydrogenase, are often present. Decreasing aminotransferase levels may indicate recovery but may also suggest worsening liver failure due to loss of hepatocyte mass. For coagulation evaluation, viscoelastic testing with rotational thromboelastography or rotational thromboelastometry should be used and is recommended over INR in critically ill patients by the Society of Critical Care Medicine, as INR reflects the extent of liver injury rather than determining coagulopathy [19]. Arterial ammonia levels have been shown to have a direct correlation with the prognosis of ALF.

## 4. Diagnosis

Identifying the etiology is important to guide the treatment and to provide prognostic information. A comprehensive medical history, with a particular emphasis on medications, and herbal and nutritional supplements, within the past six months should be performed. Urine and serum toxicology screenings, urinary ethyl glucuronide, or serum phosphatidyl ethanol should be obtained if alcohol-related liver disease is suspected. If the history, laboratory workup, and imaging findings do not provide any specific etiology for ALF, a liver biopsy might be needed; however, the risks associated with bleeding and death must be accounted for [3]. A routine liver biopsy is not recommended, and if needed, a transjugular liver biopsy is preferred as opposed to a percutaneous liver biopsy. Differentiating severe acute hepatitis from ALF mainly depends on the presence of HE. Patients with severe acute hepatitis may have INR > 1.5 but not have HE.

Differentials of very high transaminase levels (>3000 IU/L) and total bilirubin <5.0 mg/dL include mainly ischemic hepatic injury and acetaminophen (or less likely heat stroke or cocaine toxicity). Poor perfusion causing ischemic hepatic injury can be due to hypotension due to shock of any cause, heart failure, hypoxia, pulmonary failure, major surgery, trauma, and cocaine use. Neurologic Wilson disease usually includes dysarthria, dystonia, tremors, and Parkinsonism as opposed to HE secondary to ALF due to Wilson disease.

ALF can be further categorized as hyperacute, acute, and subacute based on the onset of encephalopathy according to the O’Grady classification [2]. Time frames are defined as <7 days for hyperacute ALF, 7–21 days for acute ALF, and >21 days for subacute ALF. The risk of cerebral edema is highest with hyperacute ALF and lowest with subacute ALF; however, the risk of death is inverse, being lowest with hyperacute ALF and highest with subacute ALF [2].

## 5. Imaging Findings

Abdominal Doppler ultrasonography should be obtained to evaluate for Budd–Chiari syndrome, portal hypertension, hepatic steatosis, hepatic congestion, and underlying cirrhosis. Cirrhosis may be present in patients with ALF due to Wilson disease, vertically transmitted HBV, AIH, and imaging may reveal nodular and heterogenous appearance of cirrhosis. Imaging may also reveal splenomegaly and ascites, which may be due to liver cirrhosis. It should be noted that a massively necrotic liver due to ALF may also look nodular and does not necessarily indicate cirrhosis. Pulmonary vascular congestion may be present in up to 30% of the patients with ALF on chest radiography. Neuroimaging with computed tomography (CT) or magnetic resonance imaging (MRI) may reveal cerebral edema; however, CT is not sensitive in the early stages.

## 6. Management of Acute Liver Failure and the Role of Prognostic Scores

The management of specific etiologies of ALF is shown in Table 3. Empiric therapy is often started while awaiting workup for ALF if a patient’s history provides a particular etiology, in particular for acetaminophen toxicity. N-acetylcysteine (NAC) treatment improves outcomes in patients with acetaminophen-associated ALF; however, the quality of evidence for the optimal route of administration and dosing is poor [4]. In addition, controversy exists regarding when to stop NAC, whether at the end of the standard 72 h period or with an improvement in liver tests [1]. The most commonly used endpoint is the improvement in INR <1.5, and others include ALT <50% of its peak value or three consecutive values <1000 U/L, and INR < 2, and/or undetectable acetaminophen level [2,20]. In non-acetaminophen-related ALF, NAC can be considered in the context of clinical trials; however, there are reports suggesting that IV NAC improves transplant-free survival in patients with early-stage non-acetaminophen-related ALF [21,22,23,24]. A single dose of activated charcoal can be given if acetaminophen ingestion is known to have occurred within four hours, after consideration of a patient’s level of consciousness and risk for aspiration [2]. The use of corticosteroids has not been proven to be beneficial in idiopathic DILI-related ALF [25,26].

Abnormal coagulation tests such as prolonged INR and prothrombin time do not necessarily mean an increased risk of bleeding in patients with ALF, and reports suggest that despite laboratory findings of coagulation abnormalities, most patients have a normal coagulation state [27,28]. Clinically significant bleeding is uncommon in ALF and is seen in approximately 5–10% of patients [29,30]. Prophylactic correction of platelet levels is not necessary, and routine use of fresh frozen plasma (FFP) and other coagulation factors are not supported by evidence, and in fact may lead to increased death or LT due to increased transfusion reactions, thrombosis, and transfusion-related acute lung injury [2,29,31,32]. When considering the insertion of an intracranial pressure (ICP) probe, any hemostasis abnormalities may need treatment. It is advisable to opt for a viscoelastic-test-guided (thrombelastography or rotational thromboelastometry) approach using coagulation factors such as fibrinogen concentrate or prothrombin complex concentrate (PCC). This is preferable over the use of FFP and cryoprecipitate, based on recent findings [33]. Notably, FFP is linked with transfusion-related acute lung injury/transfusion-associated circulatory overload risks and potential infections, and similar concerns arise with cryoprecipitate [34]. Furthermore, FFP displays reduced thrombin generation capability when compared to PCC [35]. An additional limitation of cryoprecipitate and FFP is the need for thawing prior to use, along with unpredictable fibrinogen levels after transfusion, which contrasts with the predictability seen when using fibrinogen concentrates [36].

The insertion of an ICP monitor is controversial and center-dependent. No changes in outcomes have been reported in patients who had ICP monitors or had more interventions to reduce ICP compared to patients who did not, although randomized clinical studies are lacking [4,31,37]. AASLD guidelines recommend ICP monitor use only in patients who are likely to have LT, while the European Association for the Study of the Liver recommends ICP monitoring in patients at high risk for intracranial hypertension (ICH) [3,38]. ICP monitoring is associated with bleeding and infection risks.

HE is essential for the diagnosis of ALF and may range from a decreased level of awareness to a deep coma. A physical exam may reveal asterixis, agitation, hyperreflexia, and clonus. Other causes of neurological disturbance must be excluded, including hypoglycemia, hypercapnia, seizures, stroke, encephalitis, and the effect of sedative medications. The complex etiology of encephalopathy, cerebral edema, and increased ICP in ALF has been thoroughly investigated in many studies. Cerebral edema may cause ICH in patients with ALF. Increased ICP is associated with a worse prognosis in patients with ALF [21]. Hyperammonemia has been shown to be associated with cerebral edema and is the key driver of astrocyte swelling due to ammonia metabolism to glutamine within astrocytes [4]. Although ammonia-lowering therapies such as lactulose or rifaximin are used for HE in the setting of ALF, there is no clear evidence of the benefit in the setting of ALF [4,31]. Younger patients (<35 years), those with high serum ammonia (150–200 µmol/L), high-grade HE (grade III, IV), SIRS or infection, and patients requiring vasopressor support or RRT have a higher risk of developing cerebral edema [39]. Intubation should be considered for patients with grade III and IV HE. Hyponatremia is often corrected as it increases cerebral edema, with sodium aimed to be between 145 and 155 mEq/L; however, no supportive evidence exists in the literature. Empiric use of treatments to reduce ICP, including hypertonic saline, l-ornithine l-aspartate (LOLA), mannitol, hyperventilation, or moderate hypothermia, has not been shown to improve mortality in ALF [2,21]. Benzodiazepines and opioids should be avoided and sedation should be minimized.

Acute kidney injury (AKI) is present in approximately 70% of patients with ALF and 30% of those patients require RRT [40]. Risk factors for AKI are increased age, acetaminophen-related ALF, hypotension, presence of SIRS, and infection. Early RRT may be considered regardless of conventional RRT criteria, especially if ammonia is high (>150 μg/dL), to avoid brain herniation [4,41,42]. Continuous modes of RRT are preferred over intermittent dialysis in patients with ALF as they avoid a wider range of metabolic and hemodynamic fluctuations associated with intermittent dialysis, which can increase cerebral edema and ICP [2,4]. Continuous renal replacement therapy (CRRT) has been shown to improve 21-day transplant-free survival rates compared to intermittent hemodialysis (HD) or no RRT; however, evidence is limited due to the lack of multiple randomized controlled trials [43].

Most patients with ALF are volume-depleted on presentation and require crystalloid volume resuscitation. In cases of persistent hypotension despite volume repletion, norepinephrine is the initial vasopressor of choice [2]. If hypotension persists despite intravenous fluid resuscitation and norepinephrine, vasopressin should be added. If there is persistent hypotension with norepinephrine plus vasopressin, hydrocortisone should be considered. If hypoxic hepatic injury is present, the use of inotropic agents should be considered.

**Figure 1:**
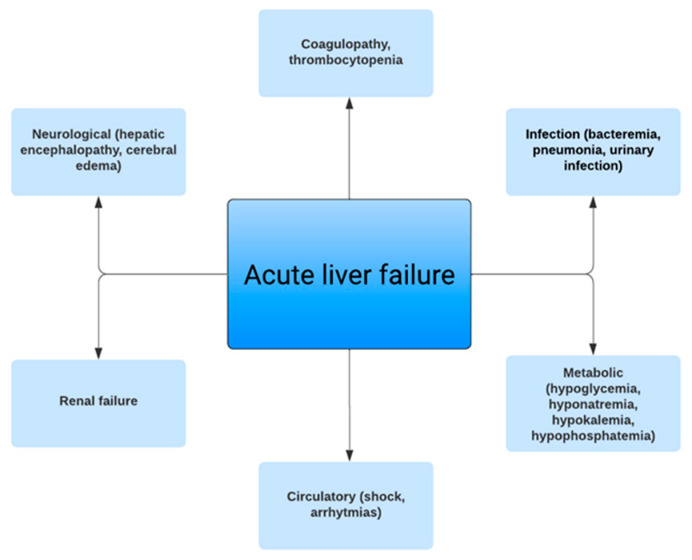
Clinical manifestations of acute liver failure.

The majority of deaths in patients with ALF are due to multiorgan failure and severe sepsis as immune dysfunction is common [11]. Routine blood, urine, and sputum cultures can aid in the early detection and treatment of infections and must be sent for all patients with ALF at admission [31]. Any deterioration in clinical status should prompt repeat cultures even if the initial cultures are negative; however, the optimal frequency is unknown [2]. Fungal infection biomarkers may have high false positive rates but low false negative rates. There is insufficient evidence for the use of procalcitonin as a marker of infection in ALF [2]. Empiric broad-spectrum antimicrobials should be considered in patients with SIRS or refractory hypotension. Prophylactic antibiotics should be considered in patients awaiting LT as those patients have an increased risk of peritransplant infection; however, the use of prophylactic antibiotics has not been shown to decrease the risk of infection in patients with ALF [2,4,44]. Figure 1 shows the clinical manifestations of ALF.

Extracorporeal liver support devices have attracted interest in the management of ALF as a bridge to transplant or liver recovery. The most commonly used artificial systems are based on plasma exchange and albumin dialysis; however, there is a lack of evidence regarding their routine use in ALF, and none of the devices have been approved by the US Food and Drug Administration [2].

Multiple prognostic scores have been developed for ALF, with the two most common being King’s College Criteria (KCC) and Model for End-Stage Liver Disease (MELD) score [2,45]. For transplant-free survival, KCC has a sensitivity and specificity of 68% and 82%, respectively, in non-acetaminophen-related ALF, and 65% and 93%, respectively, in acetaminophen-related ALF [46]. The addition of lactate to the KCC increases its sensitivity to 91% [47]. As the sensitivity of KCC is limited, concerns have been raised that it may be a poor predictor of death without transplantation. MELD >33 for acetaminophen-related ALF, and MELD >32 for non-acetaminophen-related ALF had a sensitivity of 74% and specificity of 67% [2]. Pooled data comparing KCC vs. MELD reported that KCC had lower sensitivity than MELD (59% vs. 74%) but higher specificity (79% vs. 67%) [48]. Clichy–Villejuif criteria is another criterion used mainly in Western Europe for prognostication of ALF and consists of the degrees of HE and coagulation factor V level and age [49]. However, its use is limited as factor V level testing is not widely available in most centers.

Early referral to a tertiary LT center should not be delayed while waiting for diagnostic workup and imaging test results [50]. Decreasing levels of transaminases, increasing bilirubin, and INR are poor prognostic signs and these patients must immediately be transferred to an LT center with an ICU. Even patients who may not be LT candidates should be transferred to an experienced LT center to increase their chances of survival, and should be admitted to a specialized critical care unit, as management in the ICU decreases the mortality from 80% to 30%, especially in patients with grade II HE or higher [51,52].

## 7. Role of Liver Transplantation

LT is a life-saving procedure for ALF and has decreased mortality to 20% in patients with ALF [53]. Identifying patients who will and will not improve with medical management and may need emergent LT is critical. Additionally, avoiding unnecessary LT in patients who are likely to improve with medical management is important. A very early decision may result in unnecessary LT or a very late decision may result in a missed opportunity to transplant. In patients undergoing LT, procedure-related morbidity and mortality and the need for life-long immunosuppression therapy should be considered. Certain clinical features have been shown to be associated with poor survival with medical management alone, such as the presence of altered mental status due to HE and non-liver organ failure, in particular, renal failure.

Patient selection for LT in ALF differs worldwide and there are no universally set criteria. The main type of grafts used in LT are liver grafts from a deceased donor graft; however, given the urgency of LT, marginal donors such as older donor grafts or steatotic grafts might be used. Living-donor liver transplant (LDLT) is uncommon in the USA and Europe given the availability of deceased donor liver grafts, differing from the majority of countries in Asia where LDLT is more common. In the USA, if the patient is listed, they are at highest priority, status 1A, and LDLT may be considered at centers with experience, and ABO-I (ABO-incompatible) grafts in patients who are rapidly declining [2,30]. LDLT may reduce wait times for ALF and is especially important in regions where the majority of patients undergo LDLT [25,53,54]. One-year survival rates following emergency LT for ALF are approximately 70–90% [55,56]. Patients who might be potential LT candidates should be identified early to maximize the time to find a suitable donor. The presence of irreversible brain injury is the only absolute contraindication to LT in ALF. Malignant infiltration of the liver precludes evaluation for LT as those patients are not candidates for LT. If the patient has a history of cancer or has significant hepatomegaly, liver imaging or a liver biopsy may be needed to rule out malignant infiltration [3]. Vasoplegic shock with increasing vasopressor requirements, uncontrolled acute respiratory distress syndrome, extensive mesenteric ischemia, and hemorrhagic pancreatitis are all considered relative contraindications. Multidisciplinary discussions with the transplant team including hepatology, surgery, and intensive care teams along with psychiatry/social work should be engaged in for individual patient candidacy for transplant.

## 8. Outcomes

The most important factor affecting transplant-free survival and waitlist mortality is the cause of ALF [2,57]. Favorable transplant-free survival has been reported for ALF due to acetaminophen, HAV, ischemic liver injury, and pregnancy; meanwhile, HBV, DILI, AIH, and unknown etiologies have worse transplant-free survival [4,50]. The second most important factor determining the outcome is the presence of high-grade HE. Studies have indicated that the presence of high-grade HE is a critical determinant of outcomes in ALF, emphasizing the importance of early intervention and aggressive management. Outcomes of LDLT and deceased donor LT for ALF are comparable in experienced LT centers [25,58]. However, posttransplant graft and patient survival remain lower in ALF compared to LT performed for chronic liver diseases, and the risk is higher in older recipients, older grafts, or grafts without ABO matching [5,59].

## 9. Conclusions

Diagnosis and management of ALF patients are often complex and challenging. Distinguishing ALF from ACLF is important. Early diagnosis and triage to LT centers with intensive care units are crucial to increase the chances of recovery and prolong the time for LT, if needed. With improvements in intensive care, favorable clinical outcomes have been observed in patients with ALF. As ALF is rare and heterogeneous, large multicenter trials are lacking and are challenging to conduct.

## Figures and Tables

**Table 1:** Common causes of ALF.

Viruses	Hepatitis A, B, C, D, E
	Cytomegalovirus
	Epstein–Barr virus
	Herpes simplex virus 1 and 2
	Varicella-zoster virus
	Adenovirus
	Dengue virus
Medications	Acetaminophen
	Anti-tuberculosis (Isoniazid, Rifampin, Pyrazinamide)
	Statins
	Non-steroidal anti-inflammatory drugs
	Sulfa drugs
	Phenytoin
	Carbamazepine
	MDMA (ecstasy)
	Flucloxacillin
	Ketoconazole
	Nitrofurantoin
	Immune checkpoint inhibitors
	Idiosyncratic drug reactions
Genetic/Autoimmune	Wilson disease
	Autoimmune hepatitis
Vascular	Budd–Chiari syndrome
	Veno-occlusive disease
	Ischemic hepatitis
Herbals/Nutritional Supplements	Multiple agents (Kava Kava, Hydroxycut, Ma Huang, etc.)
	Dietary and weight loss supplements
	Multivitamins
Toxins	Mushroom (most commonly Amanita phalloides)
	Carbon tetrachloride
	Yellow phosphorus
Infiltration	Breast cancer
	Small-cell lung cancer
	Lymphoma
	Colon cancer
	Melanoma
	Multiple myeloma
Pregnancy-related	Acute fatty liver of pregnancy
	HELLP syndrome
	Pre-eclamptic liver rupture
Other	Sepsis
	Partial hepatectomy
	Heat stroke
	Hemophagocytic lymphohistiocytosis

Abbreviations: ALF, acute liver failure; HELLP, hemolysis, elevated liver enzymes, low platelet count.

**Table 2:** Special patterns with ALF.

Acetaminophen toxicity	Very high AST and ALT (often >3500 IU/L)
	High INR
	Low bilirubin
Ischemic hepatic injury	Very high AST and ALT (25–250 times of upper limit of normal)
	Elevated serum LDH
Hepatitis B virus	Aminotransferase levels: 1000–2000 IU/L ALT usually > AST
Wilson disease	Coombs-negative hemolytic anemia
	Aminotransferase levels <2000 IU/L
	AST/ALT ratio >2
	Markedly low ALP (<40 IU/L)
	ALP/total bilirubin ratio <4
	Rapidly progressive renal failure
	Low uric acid levels
Acute fatty liver of pregnancy/HELLP syndrome	Aminotransferase levels <1000 IU/L
	Elevated bilirubin
	Low platelet count
Herpes simplex virus	Markedly elevated aminotransferases
	Leukopenia
	Low bilirubin
Reye syndrome/Valproate or doxycycline toxicity	Minor to moderate elevations in aminotransferase and bilirubin levels

Abbreviations: ALF, acute liver failure; ALP, alkaline phosphatase; ALT, alanine aminotransferase; AST, aspartate aminotransferase; HELLP, hemolysis, elevated liver enzymes, low platelet count; INR, International Normalized Ratio; LDH, lactate dehydrogenase.

**Table 3:** Etiology-specific management of ALF.

Etiology	Management
Acetaminophen	IV N-acetylcysteine
	Activated charcoal if ingestion is within <4 h
Drug-induced liver injury	Discontinue offending medication
	Consider N-acetylcysteine if hepatic encephalopathy
	Corticosteroids if hypersensitivity or autoimmune features (typically with minocycline or nitrofurantoin)
	Supportive care
Hepatitis B virus	Tenofovir/entecavir in reactivation of hepatitis B virus
Herpes simplex virus/varicella-zoster virus	IV Acyclovir
Cytomegalovirus	IV Ganciclovir
Mushroom poisoning	IV silybin
	IV penicillin if IV silybin is not available
Wilson disease	Continuous hemofiltration
	Plasma exchange
Autoimmune hepatitis	IV corticosteroids
AFLP/HELLP syndrome	Prompt delivery of fetus
Budd–Chiari syndrome	Anticoagulation
	Transjugular intrahepatic portosystemic shunt
	Angioplasty, stenting, or shunt creation

Abbreviations: ALF, acute liver failure; AFLP, acute fatty liver of pregnancy; HELLP, hemolysis, elevated liver enzymes, low platelet count.

## Data Availability

Data sharing is not applicable. No new data were created or analyzed in this study. Data sharing is not applicable to this article.
